# Histone deacetylase inhibitor SAHA attenuates post-seizure hippocampal microglia TLR4/MYD88 signaling and inhibits TLR4 gene expression via histone acetylation

**DOI:** 10.1186/s12868-016-0264-9

**Published:** 2016-05-18

**Authors:** Qing-Peng Hu, Ding-An Mao

**Affiliations:** Department of Pediatrics, The Second Xiang-Ya Hospital, Central South University, 139 Middle Renmin Road, Changsha, 410011 Hunan China

**Keywords:** Histone deacetylase, Seizures, Microglia, Toll-like-receptor 4 (TLR4), MYD88, Histone acetylation, SAHA

## Abstract

**Background:**

Epilepsy is a common neurological disorder characterized by recurrent unprovoked seizures. Seizure-induced TLR4/MYD88 signaling plays a critical role in activating microglia and triggering neuron apoptosis. SAHA is a histone deacetylase inhibitor that regulates gene expression by increasing chromatin histone acetylation. In this study, we investigated the role of SAHA in TLR4/MYD88 signaling in a rat seizure model.

**Results:**

Sprague–Dawley rats with kainic acid (KA)-induced seizures were treated with SAHA. The expression of TLR4, MYD88, NF-κB P65 and IL-1β in hippocampus was detected at hour 2 and 6 and day 1, 2, and 3 post seizure. SAHA pretreatment increased seizure latency and decreased seizure scores. The expression levels of TLR4, MYD88, NF-κB and IL-1β increased significantly in both activated microglia and apoptotic neurons after KA treatment. The effects were attenuated by SAHA. Chromatin immunoprecipitation assays indicated that the H3 histone acetylation levels significantly decreased while H3K9 levels significantly increased in the KA treatment group. The H3 and H3K9 acetylation levels returned to control levels after SAHA (50 mg/kg) pretreatment. There was a positive correlation between the expression of TLR4 and the acetylation levels of H3K9.

**Conclusions:**

Histone deacetylase inhibitor SAHA can suppress seizure-induced TLR4/MYD88 signaling and inhibit TLR4 gene expression through histone acetylation regulation. This suggests that SAHA may protect against seizure-induced brain damage.

## Background

Epilepsy is among the most common brain disorders [1]. Previous studies suggest that inflammation plays an important role in epilepsy development [2–6]. Seizure-induced inflammation can generate a negative feedback loop on neurons [7]. Microglia secretes cytokines (e.g. IL-1β and TNF-α) during CNS pathological processes [8].

Astrocytes and microglia express toll-like-receptor (TLR) family member proteins such as TLR3 and TLR4 [9–12]. Expressed in microglia [13–15], TLR4 is closely involved in microglia inflammation responses [16]. Thus, inhibiting TLR4-mediated microglia activation in inflammatory responses could have neuroprotective effects. It has been shown that systemic administration of kainic acid (KA) can induce epileptic seizures, which causes neuroinflammation or neuronal cell death in the hippocampus CA1 and CA3 regions in animals [17–20].

Numerous studies have shown the potential of histone deacetylase (HDAC) inhibitors as novel therapeutics for ischemic stroke [21–23], multiple sclerosis [24] and Huntington’s disease [25, 26]. Histone acetyltransferases (HATs) and HDACs reportedly determine the post-translational acetylation status of chromatin [27]. While HATs leads to a transcriptionally-active chromatin structure, HDACs deacetylate and suppress transcription [28]. Therefore, HDAC inhibitors usually reactivate silenced genes [27, 28].

TLR4 signaling leads to activation of two major signal transduction pathways: the myeloid differentiation primary response protein 88 (MyD88)-dependent and MyD88-independent pathways [29]. Previous studies have suggested a key role of MyD88-dependent signaling in developing brain injuries [29, 30]. The role of microglia TLR4/MyD88 signaling in regulating neuronal cell death and release of inflammatory cytokines following KA-stimulation is, as yet, largely unknown.

In this study, we for the first time explored the role of SAHA in TLR4/MYD88 signaling and histone acetylation of TLR4 gene in KA-induced seizures.

## Methods

### Animals

Developing male Sprague–Dawley rats (3 weeks old, weighing 60–80 g) were obtained from the Experimental Animal Center of Hunan University of Chinese Medicine (Changsha, Hunan, China). The animals were housed in conventional conditions at 22 °C, 50–60 % humidity with alternating 12 h light/12 h dark cycles. Rats were allowed free access to food and water..

### Seizure induction

Male Sprague–Dawley rats were used in this study and were randomly divided into four experimental groups: (1) control group (saline); (2) KA group; (3) “KA + 10 mg/kg SAHA”group; and (4) “KA + 50 mg/kg SAHA” group. Intraperitoneal administration of KA 15 mg/kg (Sigma, St Louis, MO, USA) was used to induce seizures. Rats were continuously monitored for seizure activity for 4 h after KA administration. Behavioral seizures were graded on the Racine scale [31]: stage 1, facial clonus; stage 2, head nodding; stage 3, forelimb clonus; stage 4, forelimb clonus with rearing; stage 5, rearing, jumping, and falling. SAHA (10 or 50 mg/kg; Sigma, St Louis, MO, USA) was injected intraperitoneally 30 min before KA injection. Seizure latency times and seizure scores were analyzed. After behavioral assessments, the rats were sacrificed with an anesthetic (1 % barbital sodium, 500 mg/kg) at different post-seizure time points (hour 2 and 6, and day 1, 2, and 3). The rats were then perfused transcardially with saline and the hippocampi were immediately dissected. Samples taken from rats sacrificed at the 2 h, 6 h, 1 d and 2 d post-seizure time points were used for Western Blot, qRT-PCR, and chromatin immunoprecipitation. The samples were preserved at −70 °C. Post-seizure rats sacrificed at the three-day mark were used to the experiment of immunohistochemistry and TUNEL staining. Their brains were removed immediately and fixed overnight using cold 4 % paraformaldehyde at 4 °C.

### Western blotting

Harvested hippocampi were washed in ice-cold PBS and lysed for 10 min using an RIPA lysis buffer. Lysates were centrifuged and supernatant collected. The protein concentration in the lysate supernatant was measured with BCA protein assays (Beyotime, Shanghai, China). Western-blot was performed according to standard procedures. Briefly, equal amounts (30 μg) of proteins were separated electrophoretically by SDS-PAGE and then transferred to 0.22 mm polyvinylidene fluoride membrane (PVDF; Millipore, Bedford, MA). The membranes were soaked in 5 % skimmed milk as blocking buffer for 2 h and then incubated with appropriate primary antibodies including mouse anti-TLR4 antibody (1:1000; Abcam), rabbit anti-MYD88 antibody (1:1000; Cell Signaling, Beverly, MA, USA), rabbit anti-NF-kB P65 antibody (1:2000; Proteintech), rabbit anti-IL-1β antibody (1:500, Proteintech) and mouse anti-β-actin (1:2000; Abcam), respectively, at 4 °C overnight. After washing with phosphate-buffered saline containing 0.1 % Tween-20 (PBST), the membranes were incubated with 5 % milk containing the corresponding peroxidase-conjugated secondary antibody (1:5000; Santa Cruz Biotechnology, CA, USA) for 1 h at room temperature. Following three washes in PBST, immunoreactive bands were visualized by enhanced chemiluminescence (ECL) (Santa Cruz, CA, USA). Band pattern was analyzed with Image Quant™ LAS 4000 (GE Healthcare, Shanghai, China).

### Quantitative (q)RT-PCR analysis

Total RNA was extracted from hippocampal tissues. Reverse transcription (RT) and Real time Quantitative polymerase chain reaction (qRT–PCR) were performed with GoScript™ Reverse Transcription kit (A5000; Promega, Madison, WI) and GoTaq^®^ qPCR Master Mix kit (A6001; Promega, Madison, WI). qPCR primers were listed in Table 1. The PCR condition was as follows: for TLR4, Denaturation: 95 °C, 45 s; Annealing: 56 °C, 45 s; Extension: 72 °C, 1 min; 30 cycles; for MYD88, Denaturation: 95 °C, 45 s; Annealing: 55 °C, 60 s; Extension: 72 °C, 1 min; 30 cycles; for NF-kB P65, Denaturation: 95 °C, 45 s; Annealing: 58 °C, 45 s; Extension: 72 °C, 1 min; 30 cycles; for IL-1β, Denaturation: 95 °C, 45 s; Annealing: 57 °C, 45 s; Extension: 72 °C, 1 min; 30 cycles; for β-actin, Denaturation: 95 °C, 30 s; Annealing: 60 °C, 30 s; Extension: 72 °C, 30 s; 26 cycles.

**Table 1:** Nucleotide sequences of primers for real time quantitative polymerase chain reaction

Gene	Forward primer	Reverse primer
TLR4	5′-GAGGTTGCTGTTCTTATTCTGAT-3′	5′-GAGTGCTGAAAGTCCAGGTATT-3′
MYD88	5′-GAAATACATACGCAACCAGCAGAAA-3′	5′-CAGATGAAGGCGTCGAAAAGC-3′
NF-kB	5′-GACCTGGAGCAAGCCATTAGC-3′	5′CGGACCGCATTCAAGTCATAGT-3′
IL-1β	5′-GTGGTATTCTCCATGAGCTTTGTA-3′	5′-CCATCTTCTTCTTTGGGTATTGTT-3
β-actin	5′-TGAGACCTTCAACACCCCAG-3′	5′-GCCATCTCTTGCTCGAAGTC-3′

### Immunohistochemistry: CD68 staining

Brain tissue was fixed in 4 % paraformaldehyde and embedded in paraffin. Hippocampal tissues were sliced into 5-μm thick coronal sections in a cryostat. After dewaxing and hydrating, sections were treated with 3 % H_2_O_2_ for 30 min. After three PBS rinses, the sections were blocked using a 0.5 % bovine serum for 1 h at room temperature. The sections were then incubated overnight at 4 °C with mouse polyclonal anti-CD68 antibody (1:100; AbD Serotec, Kidlington, UK) [32]. Sections were then incubated with a goat anti-mouse secondary antibody for 2 h followed by horseradish peroxidase (HRP)-Streptavidin for 1 h at 37 °C. After three PBS rinses, sections were reacted with a 0.025 % 3, 3′-diaminobenzidine tetrahydrochloride (DAB) solution for 5 min. Finally, sections were mounted onto gelatin-coated glass slides and air-dried. We examined the slides with a Moticam Pro microscope (Moticam, Xiamen, China). Image-Pro Plus 6.0 software (Media Cybernetics, Bethesda, MD, USA) was used to calculate the CD68 stained area. The average optical density (mean density) represented the intensity of CD68 expression and was counted in four random fields in the hippocampal CA1 region per section.

### TUNEL staining

TUNEL staining was performed using situ apoptosis detection kit (Millipore, Billerica, MA, USA). Tissue sections were treated with 3 % hydrogen peroxide to quench endogenous peroxidase activity. After adding a labeling buffer, sections were treated with terminal deoxynucleotidyl transferase (TdT) and digoxigenin dUTP for 2 h at 37 °C. Specimens were then treated with peroxidase-coupled anti-digoxigenin for 30 min at 37 °C. 3,3′-diaminobenzidine (DAB) was used to detect developing signals. Sections were counterstained with methyl green, rinsed, dehydrated, and mounted. The slides were examined by Moticam Pro microscope (Moticam, Xiamen, China). Each slice was observed using a high-power microscope on three visual fields of the hippocampus, and TUNEL positive cells were counted in each group.

### Chromatin immunoprecipitation (ChIP) assay

ChIP assays were performed with chromatin immunoprecipitation kit (Catalog No. 17-20000; Millipore, MA). Hippocampus tissue was exposed to 1 % formaldehyde to crosslink with DNA proteins, ensuring DNA co-precipitation with the protein of interest. Glycine was then added to each sample tube to quench unreacted formaldehyde. Hippocampus tissue was broken open with an SDS Lysis Buffer containing Protease Inhibitor Cocktail II. Sonication was performed to shear the chromatin to a manageable size. Generally between 200 and 1000 bp of DNA is small enough to achieve a high degree of resolution during the detection step. The average fragment size is confirmed by gel electrophoresis. Protein G Agarose (Millipore, Billerica, MA) was added to each IP tube to remove proteins, or DNA, that might nonspecifically bind to the protein G agarose. Precleared chromatin was immunoprecipitated using an anti-acetyl histone H3 and H3K9 Ab (Millipore, Billerica, MA) or rabbit IgG as a control. It was then incubated overnight at 4 °C while being rotated. Protein G Agarose was added to each IP tube and incubated for 1 h at 4 °C with rotation in order to collect the antibody/antigen/DNA complex. After four sequential washes using four different wash buffers, the protein-DNA crosslinks were reversed during incubation at 65 °C. DNA was purified to remove chromatin proteins and to prepare it for the detection step. The TLR4 gene corresponding to 5′ promoter regions -627/-437 was amplified by PCR from the sheared DNA using the forward primer 5′-ACAAACAAACAAACCCACCA-3′ and reverse primer 5′-TCCCACCTGTACTGCCTCTT-3′; GAPDH was amplified as a internal reference using the forward primer 5′-TACTAGCGGTTTTACGGGCG-3′ and reverse primer 5′-TCGAACAGGAGGAGCAGAGAGCGA-3′. PCR conditions were as follows: for TLR4, Denaturation: 95 °C, 45 s; Annealing: 58 °C, 45 s; Extension: 72 °C, 1 min; 35 cycles; for GAPDH, Denaturation: 95 °C, 30 s; Annealing: 55 °C, 45 s; Extension: 72 °C, 1 min; 30 cycles. PCR products were separated on 2 % agarose gel. Band density was measured using G: BOX Chemi XR5 Imaging System (Syngene, UK).

### Statistical analysis

All data are presented as the mean ± SD. Differences among the means were analyzed by two-tailed Student *t* test or ANOVA followed by the Bonferroni’s post hoc test. P < 0.05 was considered statistically significant.

## Results

### SAHA attenuates KA-induced seizures

**Fig. 1:**
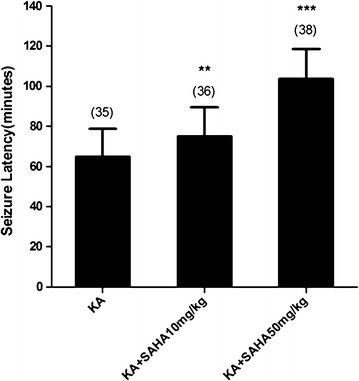
SAHA attenuated KA-induced seizures behavior in rats. The rats were pretreated with SAHA (10 or 50 mg/kg, i.p.) 30 min before KA (15 mg/kg, i.p.) injection. Seizure latency was measured during 1–4 h after KA administration. Data are expressed as mean ± SD. ***P < 0.001; **P < 0.01 or *P < 0.05 versus KA-treated group

KA induced seizure in 84 % of rats that did not receive SAHA, with 9 % mortality. Latency and seizure scores were 64.83 ± 13.99 min and 4.23 ± 0.94, respectively (Fig. 1). Different seizure scores were observed in SAHA-treated rats (Table 2). The mean latency and seizure score for “KA + 10 mg/kg SAHA” group was 75.11 ± 14.27 min and 3.44 ± 1.56, and statistically different from the seizure control group (P < 0.01 and P < 0.05; Fig. 1; Table 2). The mean latency and seizure score for “KA + 50 mg/kg SAHA” group was 103.68 ± 14.99 min and 2.37 ± 1.48, and statistically different from the seizure control group (P < 0.001; Fig. 1; Table 2). Further comparison showed that the above two data in “KA + 50 mg/kg SAHA” group were significantly different from “KA + 10 mg/kg SAHA” group (P < 0.01 and P < 0.001; Fig. 1 and Table 2). SAHA (10 or 50 mg/kg, i.p.) administration 30 min before KA administration increased the seizure latency and decreased the seizure score.

**Table 2:** Rat seizure score

	No seizure	Stage 1	Stage 2	Stage 3	Stage 4	Stage 5	Mean score
KA	0	0	3	3	12	17	4.23 ± 0.94
KA + SAHA 10 mg/kg	3	1	6	4	11	11	3.44 ± 1.56*
KA + SAHA 50 mg/kg	6	4	9	11	5	3	2.37 ± 1.48***

The rats in different seizures stage were counted and mean seizure score was analyzed by statistical method. Data are expressed as mean values ± SD

* P < 0.05; *** P < 0.001, as compared with the KA-treated group

### SAHA reduces KA-induced neuronal cell death

**Fig. 2:**
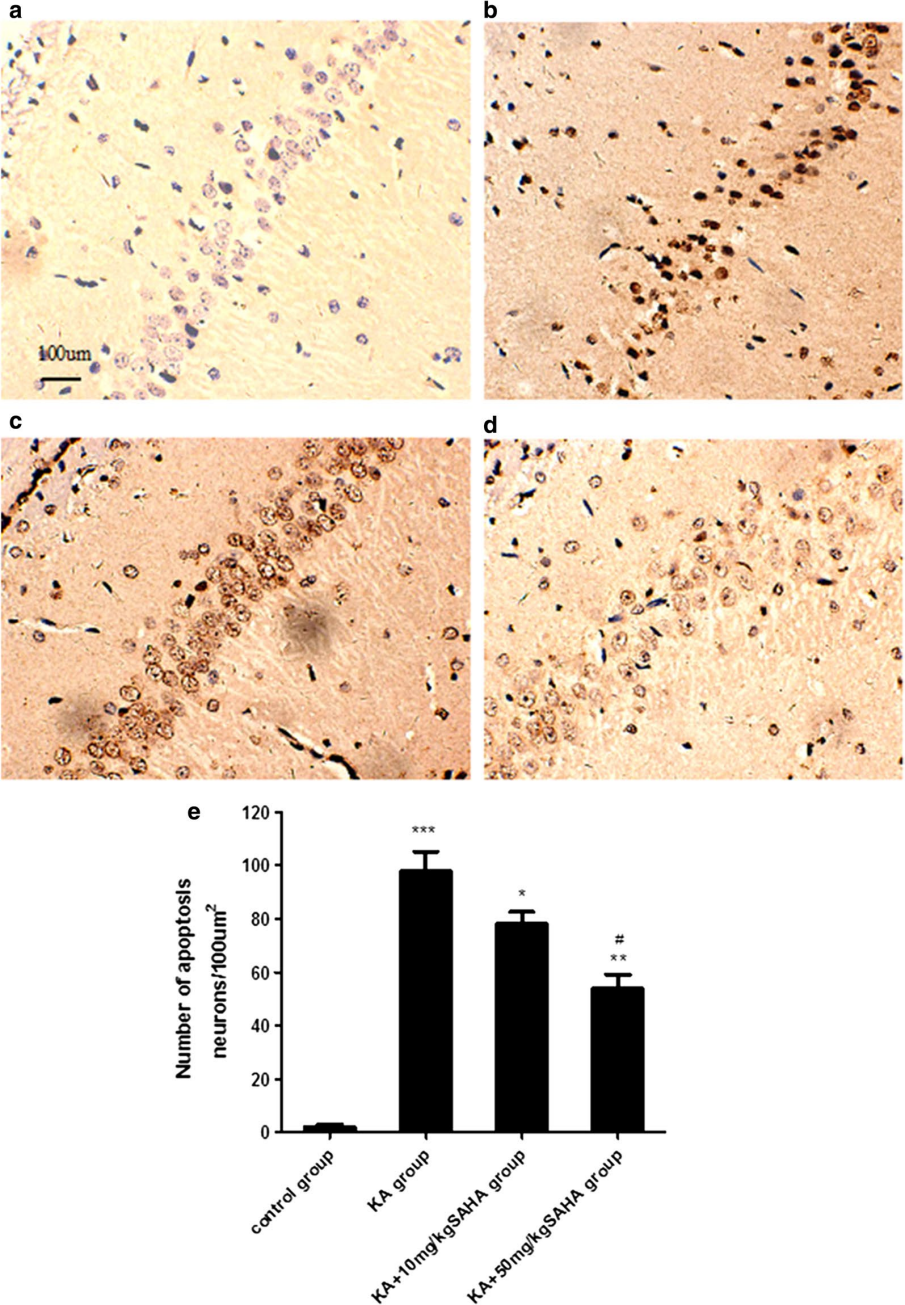
SAHA attenuated KA-induced neuronal death in hippocampal CA1 region. SAHA (10 or 50 mg/kg) was administrated intraperitoneally 30 min before KA injection. Neuronal apoptosis were evaluated 3 days after KA injection. (**a**–**d**) TUNEL staining of hippocampal CA1 pyramidal neurons; **a** control group, **b** KA group, **c** KA + 10 mg/kg SAHA group, **d** KA + 50 mg/kg SAHA group, **e** TUNEL positive cells quantities for each group. Data are expressed as mean ± SD. *Scale bar* = 100 μm. *P < 0.05, KA group versus KA + 10 mg/kg SAHA group; **P < 0.01, KA group versus KA + 50 mg/kg SAHA group; ***P < 0.001, KA group versus control group; ^#^P < 0.001, KA + 10 mg/kgSAHA group versus KA + 50 mg/kg SAHA group

TUNEL staining showed considerable neuronal apoptosis at hippocampal CA1 in the KA-injected rats (Fig. 2b). SAHA pretreatment (10 or 50 mg/kg) significantly attenuated KA-induced neuronal apoptosis (Fig. 2c, d). Figure 2a was the negative control group. In rats pretreated with SAHA 50 mg/kg, the number of TUNEL positive cells at CA1 decreased significantly in comparison to SAHA 10 mg/kg.

### SAHA suppresses KA-induced microglia activation

**Fig. 3:**
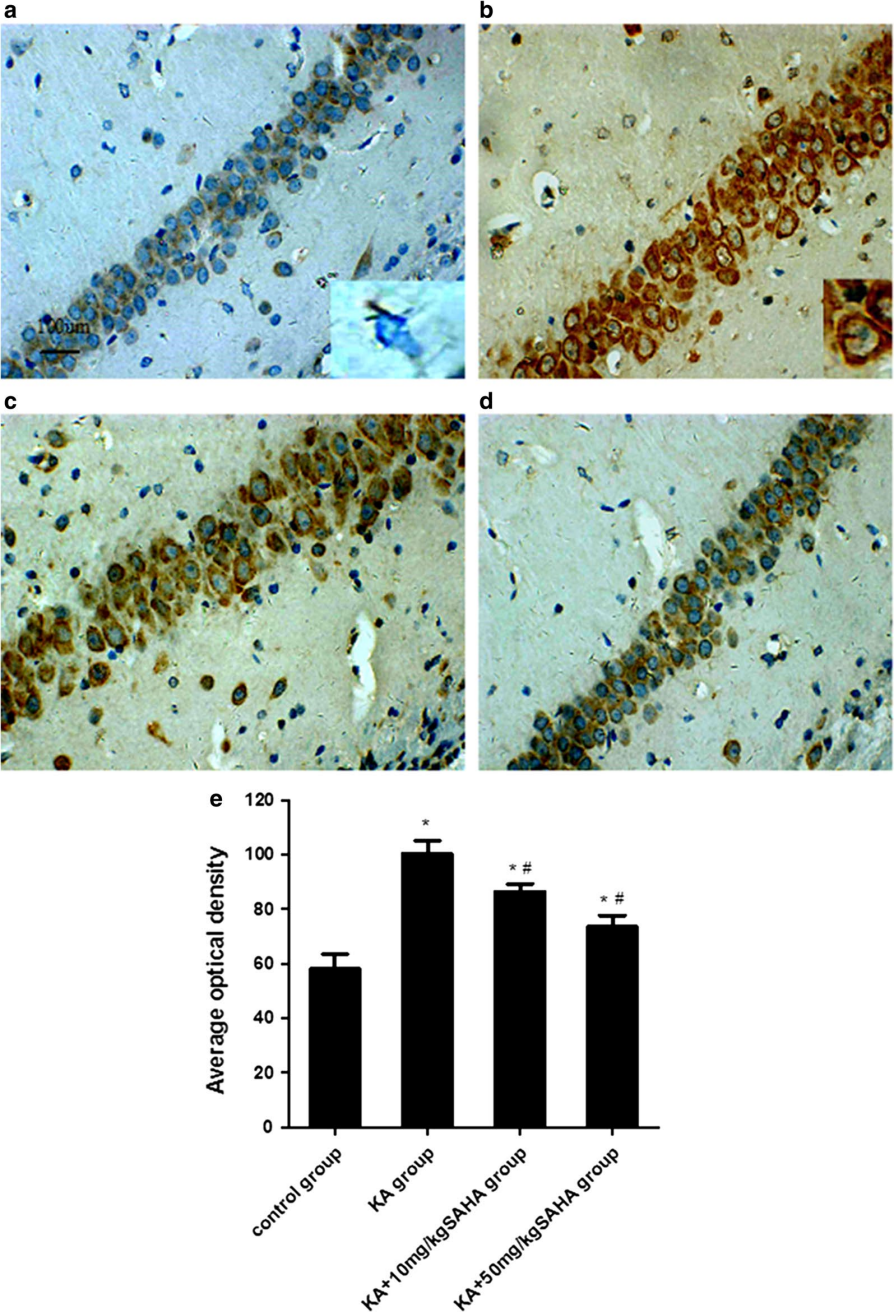
SAHA attenuated KA-induced microglia activation in the hippocampal CA1 region. SAHA (10 or 50 mg/kg) was administrated intraperitoneally 30 min before KA injection, and the hippocampal sections were stained with anti-CD68 antibody 3 days after KA injection. (**a**–**d**) CD68 immunostaining of activated microglia in hippocampal CA1 region; **a** control group; **b** KA group; **c** KA + 10 mg/kg SAHA group; **d** KA + 50 mg/kg SAHA; **e** Average optical density of CD68 immunoreactive cells in each group. *Insets* in **a** and **b** show morphological change after KA administration under higher magnification. Data are expressed as mean ± SD, *Scale bar* = 100 μm for **a**–**d**. *P < 0.01 versus control group; ^ #^P < 0.01 versus KA group

To examine whether SAHA affected inflammatory processes in KA-injected rats, we analyzed microglia activation by detecting the expression of CD68, a marker of activated microglia. CD68 positive cells and intensity increased in KA-treated rats, but this increase was suppressed by SAHA pretreatment (Fig. 3c, d). In the saline and SAHA (50 mg/kg)-treated rats, CD68 positive cells were sparse and CA1-region microglia showed a resting morphology with small cell bodies and long, thin, processes (Fig. 3a). KA-injected rats had a notable increase in the number of activated microglia in the CA1 region. These cells displayed enlarged amoeboid morphology and fewer and shorter processes, which indicated the activation state (Fig. 3b). SAHA-pretreatment significantly suppressed KA-induced microglia activation (Fig. 3c, d).

### SAHA reduces mRNA and protein levels of TLR4, MYD88, NF-κB P65 and IL-1β

**Fig. 4:**
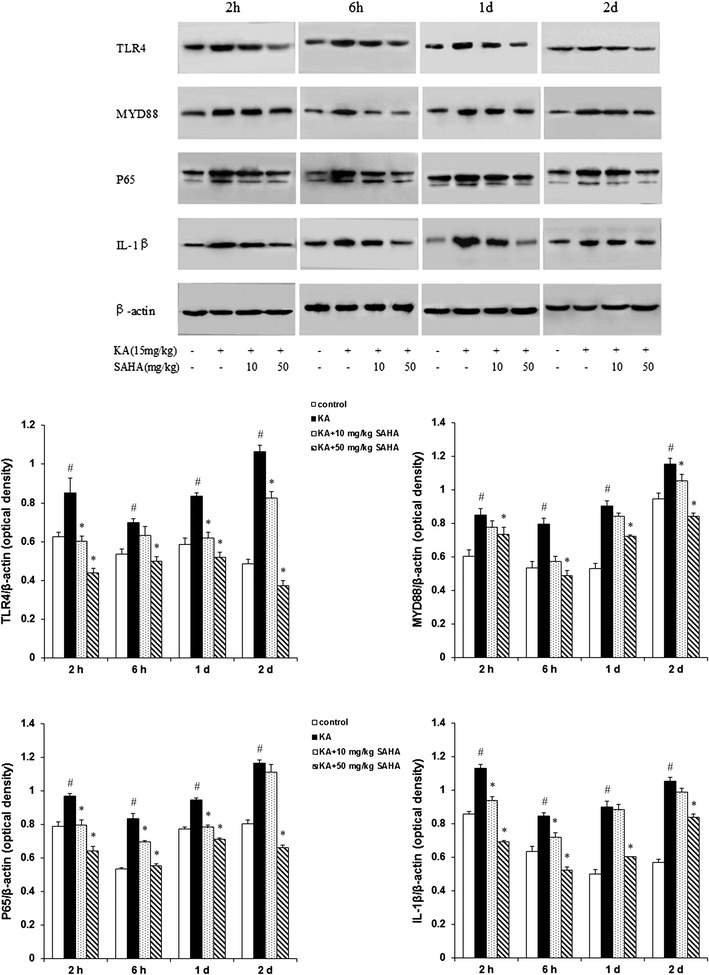
SAHA inhibited TLR4 signal pathway in KA-induced seizures. The protein expression of TLR4, MYD88, NF-κB P65 and IL-1β were examined by immunoblots at 2, 6 h, 1 d and 2 d after KA injection in hippocampus CA1 region. Specific bands were quantified by densitometry and data were expressed as mean ± SD (n = 3); ^#^P < 0.01 versus control group; *P < 0.01 versus KA-treated group

**Fig. 5:**
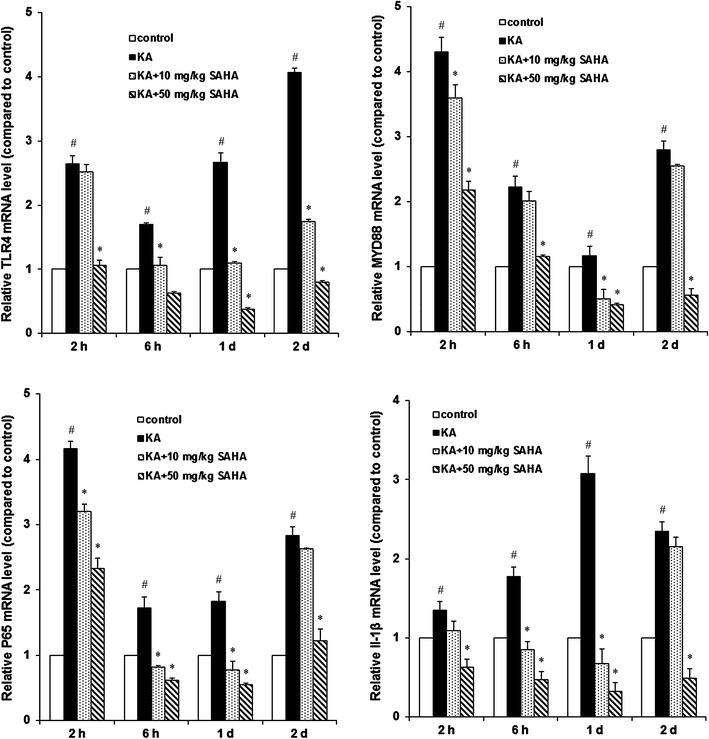
SAHA attenuated KA-induced mRNA expression of TLR4, MYD88, NF-κB and IL-1β in hippocampus. In hippocampus CA1 region, mRNA expression of TLR4, MYD88, NF-κB and IL-1β were measured with real-time Quantitative PCR at 2, 6 h, 1 d and 2 d after KA injection. Relative quantification data were expressed as mean ± SD (n = 3). ^#^P < 0.01 versus control group; *P < 0.01 versus KA-treated group

To investigate the effect of SAHA on TLR4/MYD88 signaling, Real-time qPCR and Western blot were used to measure gene expression and protein levels of TLR4, MYD88, NF-κB P65 and IL-1β. KA stimulation increased TLR4, MYD88, NF-κB P65 and IL-1β protein levels at 2 h, 6 h, 1 d and 2 d after KA injection in hippocampus CA1 region. SAHA 50 mg/kg significantly attenuated the increases (Fig. 4). Further gene expression analyses by Real-time qPCR confirmed that TLR4, MYD88, NF-κB P65, and IL-1β mRNA levels were consistent with protein level tendencies (Fig. 5). These results suggested that SAHA could suppress seizures-induced TLR4/MYD88 signaling.

### SAHA inhibits TLR4 gene transcription by histone acetylation regulation

**Fig. 6:**
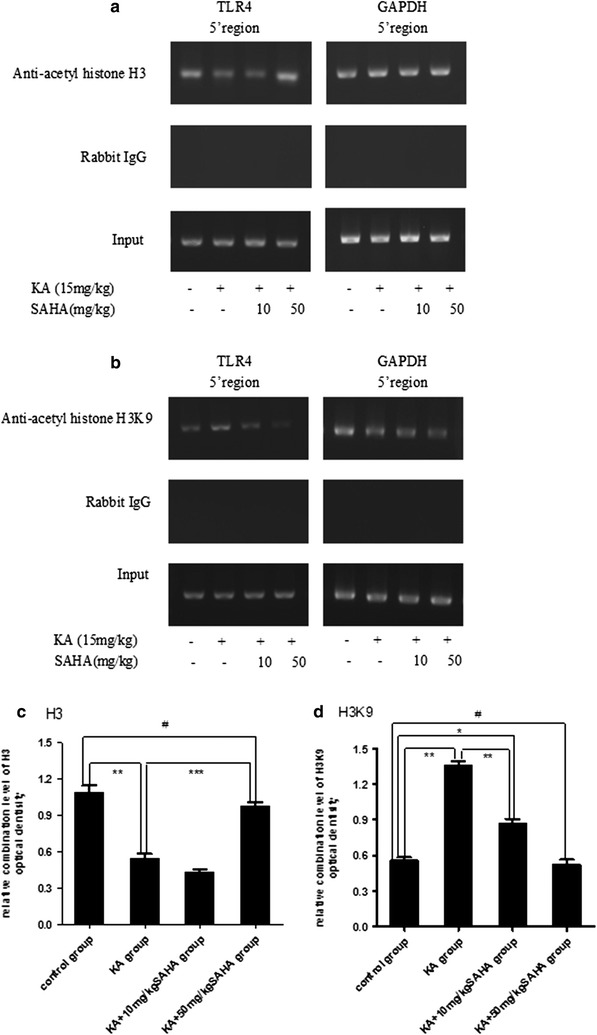
SAHA inhibited TLR4 gene transcription by histone acetylation regulation. Histones interaction with the 5′ region of the TLR4 gene were examined with ChIP assays using anti-acetyl histone **a** H3 and **b** H3K9 antibodies. Rabbit IgG was used as a negative control. The “input” *lanes* represent the results of PCR using non-immunoprecipitated chromatin as templates. The 5′ regions of the TLR4 gene, with the 5′ regions of the GAPDH gene as an internal reference, were amplified by PCR from the immunoprecipitated chromatin. The DNA bands for **c** H3 and **d** H3K9 were quantified by densitometry and data expressed as mean ± SD (n = 3). *Bars* expressed as the ratio of optical density (OD) of the samples to the corresponding internal standard (GAPDH). *P < 0.05; **P < 0.01; ***P < 0.001; ^#^P > 0.05

Twenty-four hours after KA-induced seizures, histone acetylation on the TLR4 gene 5′ region was analyzed by ChIP assays using anti-acetyl histone H3, and H3K9 antibodies. As shown in Fig. 6, H3 histone acetylation levels significantly decreased while H3K9 significantly increased in the KA treatment group when compared to the control group. H3 and H3K9 acetylation level returned to control levels after SAHA 50 mg/kg pretreatment (Fig. 6a–d). The mRNA expression of TLR4 increased significantly with KA treatment, which was reversed by SAHA (Fig. 5a). The results suggested that the expression of TLR4 was dependent on the acetylation levels of H3K9; SAHA led to hypoacetylation of H3K9, which in turn resulted in downregulation of the expression of TLR4.

## Discussion

To date, the understanding of potential protective mechanisms and pathogenesis of seizures-induced brain injury is still very limited [33–35]. We hypothesized that SAHA would have an anticonvulsant effect. This study shows that SAHA has an anticonvulsant effect in a KA seizure model in rats. We chose this model because KA-induced seizure activity and brain damage are similar to those in humans [17, 36, 37] and are associated with excessive glutamate release [38–40]. In agreement with previous studies, KA injection (15 mg/kg, i.p.) induced epileptic seizures in this study [36, 41–43]. SAHA pretreatment (10 or 50 mg/kg) reduced KA-induced neuronal apoptosis, suggesting SAHA as a potent neuroprotective and anticonvulsant agent.

Previous studies suggest that KA-induced microglia activation can cause hippocampal neuron apoptosis [44–46]. Therefore, preventing microglia activation protects hippocampus against neuron apoptosis caused by KA-induced seizures [47]. In this study, we noted that the number of activated microglia immunostained for CD68, a marker for microglia activation [48], was significantly higher in the hippocampus in KA-treated rats. SAHA pretreatment reduced the number of activated microglia. Thus, SAHA may have an anti-inflammatory function and neuroprotective effects. However, how SAHA affects microglia activation remains unclear. Microglia activation and subsequent production of pro-inflammatory cytokines are believed to contribute to neuronal damage [49, 50]. Thus, there might be a direct interaction between SAHA and these factors; this needs to be explored in future studies.

A previous study reported that a functional TLR4/MyD88 cascade in microglia was essential for neuronal injury [51–55]. In this study, our findings indicate that SAHA attenuated the inflammatory mediators production by inhibiting the TLR4/MyD88 signaling pathway associated with activated microglia. Thus, the anti-neuroinflammation effect of SAHA on activated microglia may contribute to the treatment of conditions relating to inflammatory response.

In this study, ChIP experiments indicated that KA increased histone H3 acetylated at lysine 9 (H3K9) of TLR4 gene and SAHA pretreatment reversed the increase. Lysine acetylation is a common post-translational modification on both histones and non-histones; hyperacetylation of lysine is conventionally associated with enhanced gene expression [56]. This means that TLR4 gene transcription is epigenetically suppressed by adding SAHA in KA-induced seizures, as found in this study. In agreement with previous studies showing that HDAC inhibitors increased gene expression and reversed the reduction of histone H3 acetylation levels in rat neuroinflammation and brain damage models [57, 58], we found in this study that SAHA suppressed H3K9 acetylation levels and then reduced TLR4 gene expression in the rat seizures model.

## Conclusions

In summary, the study shows that SAHA can suppress seizure-induced microglia activation and neuron apoptosis, and inhibit TLR4 expression through histone acetylation regulation by inhibiting TLR4/MYD88 signaling. This strongly suggests a potential neuroprotective effect of SAHA against neuroinflammation-induced brain damage. These findings provide new insights into the treatment of epilepsy and other neurodegenerative disorders.

## Abbreviations

ChIP: chromatin immunoprecipitation
HAT: histone acetyltransferase
HDAC: histone deacetylase
MyD88: myeloid differentiation primary response protein 88
PRR: pattern recognition receptor
TLR: toll-like-receptor
